# Inhibitory Activity of the Isoflavone Biochanin A on Intracellular Bacteria of Genus *Chlamydia* and Initial Development of a Buccal Formulation

**DOI:** 10.1371/journal.pone.0115115

**Published:** 2014-12-16

**Authors:** Leena Hanski, Natalja Genina, Hanna Uvell, Kristina Malinovskaja, Åsa Gylfe, Timo Laaksonen, Ruzica Kolakovic, Ermei Mäkilä, Jarno Salonen, Jouni Hirvonen, Mikael Elofsson, Niklas Sandler, Pia M. Vuorela

**Affiliations:** 1 Division of Pharmaceutical Biosciences, CDR, Faculty of Pharmacy, University of Helsinki, Helsinki, Finland; 2 Pharmaceutical Sciences Laboratory, Department of Biosciences, Abo Akademi University, Turku, Finland; 3 Laboratories for Chemical Biology, Umeå University, Umeå, Sweden; 4 Division of Pharmaceutical Chemistry and Technology, Faculty of Pharmacy, University of Helsinki, Helsinki, Finland; 5 Department of Clinical Microbiology, Umeå University, Umeå, Sweden; 6 Umeå Centre for Microbial Research, Umeå University, Umeå, Sweden; 7 Laboratory for Molecular Infection Medicine, Umeå University, Umeå, Sweden; 8 Laboratory of Industrial Physics, Department of Physics and Astronomy, University of Turku, Turku, Finland; 9 Department of Chemistry, Umeå University, Umeå, Sweden; Universite de la Mediterranee, France

## Abstract

Given the established role of *Chlamydia* spp. as causative agents of both acute and chronic diseases, search for new antimicrobial agents against these intracellular bacteria is required to promote human health. Isoflavones are naturally occurring phytoestrogens, antioxidants and efflux pump inhibitors, but their therapeutic use is limited by poor water-solubility and intense first-pass metabolism. Here, we report on effects of isoflavones against *C. pneumoniae* and *C. trachomatis* and describe buccal permeability and initial formulation development for biochanin A. Biochanin A was the most potent *Chlamydia* growth inhibitor among the studied isoflavones, with an IC_50_ = 12 µM on *C. pneumoniae* inclusion counts and 6.5 µM on infectious progeny production, both determined by immunofluorescent staining of infected epithelial cell cultures. Encouraged by the permeation of biochanin A across porcine buccal mucosa without detectable metabolism, oromucosal film formulations were designed and prepared by a solvent casting method. The film formulations showed improved dissolution rate of biochanin A compared to powder or a physical mixture, presumably due to the solubilizing effect of hydrophilic additives and presence of biochanin A in amorphous state. In summary, biochanin A is a potent inhibitor of *Chlamydia* spp., and the in vitro dissolution results support the use of a buccal formulation to potentially improve its bioavailability in antichlamydial or other pharmaceutical applications.

## Introduction

Biochanin A is the main isoflavone component of red clover (*Trifolium pratense* L.) and the commercially available extracts made of this plant [1]. These botanical dietary supplements are sold in tablet form in several countries to alleviate postmenopausal symptoms in women. In contrast to its unmethylated analogue genistein, biochanin A is not present in soy at significant quantities, but it can be found in many other legume plants and peanuts. The role of biochanin A and other isoflavones in these plants is not known in detail, but generally speaking such secondary metabolites are produced to protect the plant from radiation and microbial attacks.

Most beneficial health effects linked to isoflavones such as biochanin A are believed to be mediated by the estrogenic and antioxidative properties of these compounds. Epidemiological studies have demonstrated the protective effects of isoflavone-rich diets against breast and prostate cancer [2 and references therein], and the affinity of isoflavones to estrogen receptors is thought to mediate also their osteoprotective effects [3]. According to different *in vitro* studies, genistein is more potent and efficient than biochanin A in terms of both estrogenic and antioxidant activity [4], [5]. However, biochanin A is efficiently converted to genistein during first pass metabolism, and genistein can be detected from human plasma after ingestion of biochanin A [6].

Despite their various biological activities described *in vitro* and in several animal and human studies, intake of isoflavones is known to involve a major pharmacokinetic problem related to their poor bioavailability and significant interpersonal variation [6], [7]. One major factor in the compromised oral bioavailability of these compounds is their poor water-solubility; for instance biochanin A has a reported solubility of 7 µg/ml in water [8]. Another important contributing factor for flavonoids' poor bioavailabilities is their extensive metabolism and participation in enteric and enterohepatic recycling processes, which limit their entry to systemic circulation. Due to its high permeability, biochanin A has been shown to rapidly and efficiently penetrate into Caco-2 cells and to be absorbed in rat intestinal perfusion models [9]. However, the absorbed biochanin A is efficiently conjugated by different UGT and sulphatase isoforms in enterocytes and hepatocytes and the conjugates are excreted to intestinal lumen both directly (from the enterocytes) and indirectly (from the hepatocytes via bile production) [9]. The conjugates can be further hydrolysed by intestinal microbes and biochanin A and other flavonoids may thus undergo extended recycling instead of reaching the systemic circulation.

Besides the phase II metabolism reactions, biochanin A and other methylated isoflavones undergo demethylation reactions in liver by phase I metabolizing enzymes [9], [10] It has been suggested that CYP1A2 is the main cytochrome P450 isoform in human liver responsible for conversion of methylated isoflavones to genistein and daidzein via the *O*-demethylation pathway as well as for the further metabolites of genistein to 3-OH-genistein [10]. As discussed above, this is seen in the appearance of genistein in bloodstream after oral intake of pure biochanin A, while typically less than 5% of biochanin A is absorbed in its parent form [6].

The intracellular bacteria in family *Chlamydiaceae* include human pathogens with high prevalence in both Western world and developing countries. *Chlamydia trachomatis* is a sexually transmitted bacterium replicating in mucosal epithelium and being among the most common sexually transmitted diseases worldwide [11]. Ocular *C. trachomatis* strains infect also conjuctival epithelial cells, which may translate to trachoma, the most common form of preventable infectious blindness [12]. On the other hand, *C. pneumoniae* is an air-borne respiratory tract pathogen responsible for 5–10% of community acquired pneumonias and causing upper and lower respiratory tract infections with varying severity [13], [14].

Besides epithelial cells, these unique Gram-negative bacteria can infect at least smooth muscle cells, fibroblasts, and peripheral blood mononuclear cells (PBMC), and particularly *C. pneumoniae* and *C. trachomatis* LGV strains have been repeatedly isolated from body sites distant from the primary infection [15]–[17]. Both *C. pneumoniae* and *C. trachomatis* have been widely studied for their propensity to convert to a persistent form of infection, which is difficult to eradicate with currently available medication [18], [19]. Based on these and other findings, they have been extensively discussed in the context of microbial burden as a risk factor for several chronic diseases [20], [21].

Thus, *C. pneumoniae* and *C. trachomatis* represent Gram-negative bacteria with major treatment challenges due to their propensity to persistence and compromised susceptibility to first-choice antibiotics. We have previously conducted early drug discovery applying a spectrum of different strategies with a hypothesis that identifying novel types of small molecule inhibitors against these pathogens provides means for more effective treatment strategies and sheds light on the molecular details of the chlamydial infections [22]–[25].

In the current work, we demonstrate that biochanin A, a naturally occurring methylated isoflavone, is a potent inhibitor of the growth of *C. pneumoniae* and *C. trachomatis.* In an attempt to avoid the extensive first pass metabolism associated with peroral delivery of biochanin A, permeation through porcine buccal mucosa was studied and different film formulations for buccal delivery were developed and investigated with regard to their solid-state characteristics and *in vitro* dissolution rates.

## Materials and Methods

### Materials

**Figure 1:**
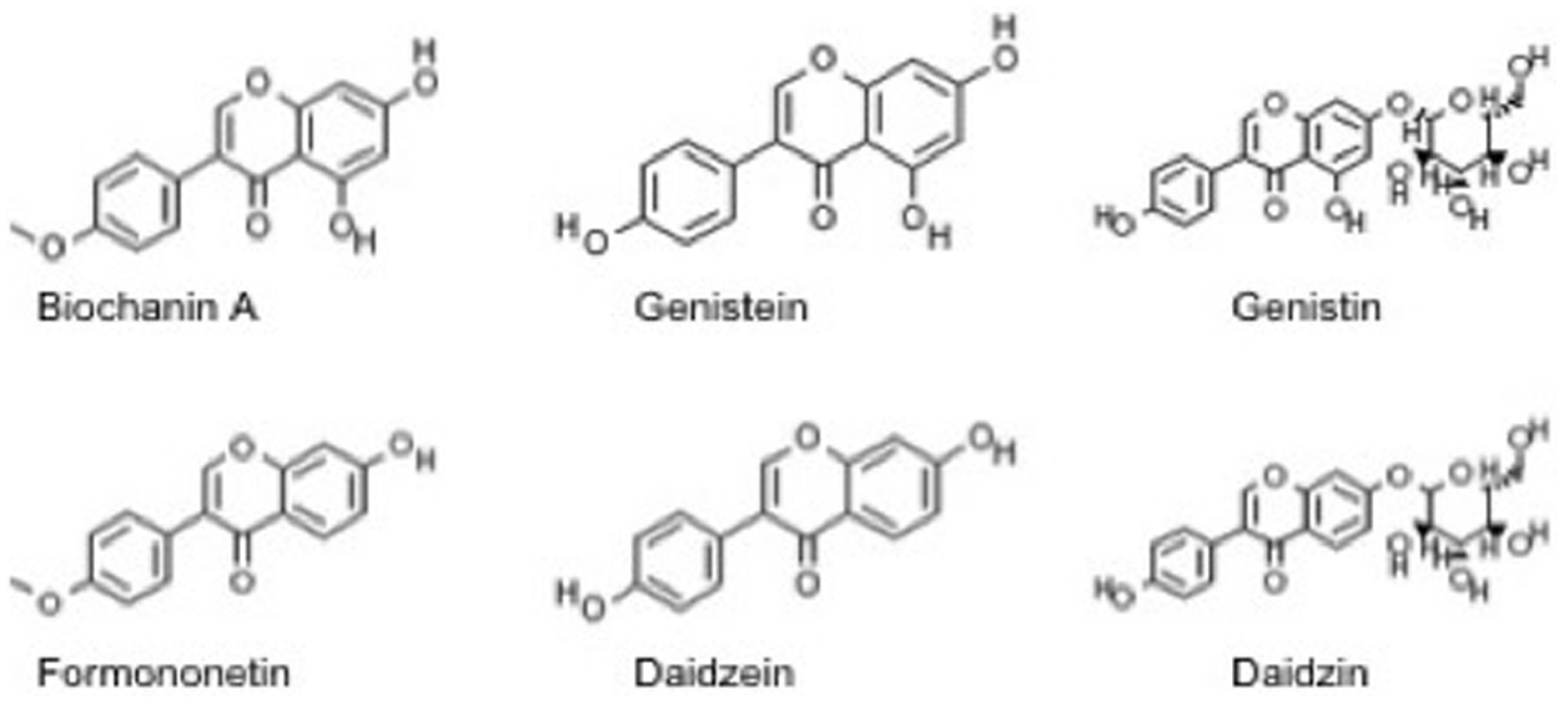
Chemical structures of the six isoflavones included in the study.

Biochanin A, formononetin, genistein, daidzein, genistin and daidzin (Fig. 1) were purchased from Sigma-Aldrich, (St. Louis, MO, USA) and dissolved in dimethyl sulfoxide (DMSO) for the biological experiments. The purity of the compounds were >98.0%. For formulation studies, biochanin A was dissolved in ethanol. Rifampicin used as a positive control in experiments with *C. pneumoniae* was from Fluka (Buchs, Switzerland) and was dissolved in ethanol. Hydroxypropyl cellulose (HPC, Klucel LF), crospovidone and sodium starch glycolate were provided by Astra Zeneca, Sweden. Hydroxypropyl methylcellulose (HPMC, Metolose 90SH-4000) and sodium dodecyl sulfate (SDS, Fluka Biochemika) were purchased from Japan. Polyvinylpyrrolidone (PVP, K-30, Mr∼40000, Switzerland) and Tween 20 were obtained from Fluka Chemicals. Maize starch (C*Gel, Cargill) was obtained from Segerha. Propylene glycol (PG) was purchased from Germany. A flavoring agent levomenthol was obtained from Yliopiston Apteekki, Finland. All other chemicals and solvents used were of analytical grade.

### Cell culture and Chlamydia stocks

Human epithelial HL cells of respiratory tract origin [26] were grown in RPMI 1640 (BioWhittaker, Lonza, Basel, Switzerland), supplemented with 7.5% fetal bovine serum (FBS) (BioWhittaker, Lonza, Basel, Switzerland), 2 mM L-glutamine (BioWhittaker, Lonza, Basel, Switzerland) and 20 µg/ml of gentamicin (Fluka, Buchs, Switzerland). HeLa-229 cells (CCL-2.1; ATCC, Manassas, VA, USA) were maintained in RPMI1640 supplemented with 20 mM HEPES (pH 8.0), 10% FBS, 2 mM L-glutamine, 8 µg/ml gentamicin and 1 µg/ml amphotericin B. The cells were cultured in standard cell culture protocols at 37°C and 5% CO_2._ For all infections with *C. pneumoniae*, HL cells were seeded into 24-well plates with coverslips at density 4×10^5^ cells per well and incubated overnight before used for infection.

The *C. pneumoniae* clinical isolate K7 [27] was propagated as described in Alvesalo *et al.* [23] and stored in SPG buffer in −70°C. *C. trachomatis* serovars K (VR-887, ATCC) and L2 (VR-902B; ATCC) were propagated in HeLa 229 cells and handled as described in [28].

### Infections

*C. pneumoniae* infections in HL cells were carried out by diluting the bacterium in the HL cell growth medium supplemented with 1 µg/ml cycloheximide (Sigma-Aldrich, St. Louis, MO, USA) and inoculating HL cell monolayers with the multiplicity of infection (MOI) 0.2. After inoculation, the plates were centrifuged at 550×g for 1 h at 4°C and then incubated at 37°C for 1 h. Then the inocula were removed and fresh medium with assayed compounds or solvent control (DMSO 0.2–0.5% depending on experiment) was added. At 72 h post infection, the wells were washed with PBS and the coverslips were fixed with methanol. The staining of host cells and chlamydia inclusions was carried out using Pathfinder *Chlamydia* Culture Confirmation System reagent (Bio-Rad, Hercules, CA, USA) and the inclusion counts were determined under fluorescent microscope. *Chlamydia trachomatis* infections in HeLa-229 cells were done essentially as described in [28], with the following exceptions; the concentrations tested were 100, 75, 50, 25, 10, 5 and 2.5 µM and the HeLa cells were infected 18 h before fixation and immunostaining. The immunostainings were analyzed using an ArrayScanVTI HCA reader (Thermo Fisher Scientific Inc., USA). Rifampicin was used as a positive control in the infections, consistently yielding 95–98% reduction in inclusion counts at concentration 12 nM.

### Infectious progeny assay

To assay the effect of the compounds on the production of infectious progeny, HL cells were infected with *C. pneumoniae* and treated with the compounds as described above. At 72 h, two coverslips were fixed and stained as described above to confirm the infectivity and inhibitory effects of the compound. In another two wells medium was removed, 200 µl fresh medium was added and cells were scraped off and ruptured by vortexing with glass beads. The solution was used to infect fresh monolayers of HL cells seeded the day before. At 144 h, the cultures with the second passage infections were fixed and stained as described above.

### Pretreatment experiments

For *Chlamydial* elementary body (EB) pretreatment experiments, *C. pneumoniae* stocks were diluted in the infection medium and biochanin A was added in aliquots of the diluted EB suspension. The suspension was incubated at 4°C for 1 h and was then used to infect HL cells as described above. For host cells pretreatment experiments, HL cells seeded in 24-well plates were incubated in the presence of biochanin A at 37°C for 1 h. Then, the samples were removed, the cell monolayers were washed once with infection medium and infected with *C. pneumoniae* as described above.

### Cell viability assays

HL and HeLa cells were seeded into 96-well plates at density 6×10^5^ cells per well and incubated overnight before exposed to the compounds in various concentrations for 72 h. The medium was removed after the exposure period and 20 µM resazurin solution in PBS was added into the wells. The plates were incubated for 2 h at 37°C and the fluorescence was recorded at 570/590 nm (ex/em).

### Preparation of buccal films and physical mixture

Buccal films of water-soluble polymers HPC and HPMC were prepared by solvent casting method. HPC-containing films were prepared using ethanol as a solvent, whereas HPMC films were prepared from aqueous-alcoholic solutions. The composition of different formulations is presented in Table 1. Alcoholic and aqueous solutions were prepared by dissolving the polymer (%w/w) and the plastisizer in the solvent and allowing it to stir for 2 h. After that all excipients were added (except for formulations Fjj, Fkk, Fll) and the solutions/suspensions were stirred for 2 h. When the maize starch was used (Fbb, Fjj, Fkk and Fll), the suspension was heated above the gelation point of starch (64°C) at 70°C for 30 min and after that continued stirring for 1.5 h. The amount of 5 mg/ml biochanin A powder (except for Fii) was added to all formulations. Formulation Fii was prepared by first dissolving biochanin A into pure alcohol and then adding 2% HPMC aqueous solution to get 5 mg/ml biochanin formulation. In addition, formulations containing 2.5 mg/ml and 10 mg/ml biochanin A were prepared (composition corresponding to formulation Faa). In the case, when solubilizers were used (Fjj, Fkk, Fll), biochanin A was first premixed with them and after that the liquid formulations were added aiming to prepare 5 mg/ml mixtures. The biochanin A-containing preparations were stirred until the isoflavone was dissolved. After that the formulations were kept unstirred for 0.5 h to remove all the bubbles entrapped. The formulations were cast onto intermediate liners (DatalineTM Transparency film, code 57167, EU) with 1 ml of the formulation per 10 cm^2^ of the liner, with the aim to produce 4 cm^2^ cut squares (2×2 cm) containing 2 mg of the drug in each. The films were left to dry in ambient conditions (relative humidity 45±5%, temperature 22±2°C) for 12 h. The produced films were stored in tightly closed folium envelopes until further analysis. The physical mixtures corresponding to formulation Faa were prepared by using spatula and mortar before analysis.

**Table 1:** Composition of sublingual films of biochanin A.

Ingredients	Faa	Fbb	Fdd	Fee	Fii	Fjj	Fkk	Fll
Biochanin A (mg/ml)	5	5	5	5	5	5	5	5
HPC (%w/w)	5	5	5	5	—	5	5	5
HPMC (%w/w)	—	—	—	—	1	—	—	—
PVP, K-30**	—	—	—	—	—	—	—	1∶2
SDS**	—	—	—	—	—	—	1∶2	—
Tween 20**	—	—	—	—	—	1∶2	—	—
Crospovidone*	—	—	—	16.7	—	—	—	—
Na starch glycolate*	—	—	16.7	—	—	—	—	—
Maize starch*	—	16.7	—	—	—	16.7	16.7	16.7
Propylene glycol (PG)*	6.3	6.3	6.3	6.3	5.9	6.3	6.3	6.3
Levomenthol*	—	—	—	—	—	0.7	0.7	0.7

*Quantities are expressed in %w/w of polymer; **Quantities are expressed in ratio of biochanin A to excipient.

### *In vitro* dissolution studies

The dissolution testing was performed according to the USP paddle method by using the Sotax AT7 Smart dissolution tester (SOTAX, Switzerland) and UV/Vis spectrophotometer (PerkinElmer, Lambda 25, USA) at 261 nm. In order to maintain sink conditions and mimic the pH of saliva, the dissolution experiments were carried out in 900 ml of phosphate buffer at pH 6.8 (0.1 M NaH_2_PO_4_·H_2_O and Na_2_HPO_4_·2H_2_O in water) at rotation speed of 100 rpm and temperature of 37±0.5°C. The films of ∼4 cm^2^ were put into the spiral capsule sinkers to prevent floating. Powder and physical mixture were immersed into the dissolution media at concentration, corresponding to 2 mg of biochanin A per vessel.

### Thickness

The thickness of each film was measured using a Digimatic caliper (AbsoluteTM Digimatic, Mitutoya Corporation, Kawasaki, Japan) at four locations.

### Optical microscopy

Optical microscopy (OM, Evos XL, AMG, USA) in connection with a digital camera (DC) was used to visualize the samples at magnifications of 4× and 40×.

### Folding endurance

Folding endurance was determined by bending the films at the same place until it broke. The number of times the films folded without breaking was counted.

### X-ray diffraction (XRD)

The X-ray diffraction patterns of the film samples were measured with an X-ray diffractometer (Philips, X'Pert PRO MPD, Holland). Measurements were performed in θ/2θ Bragg-Bretano geometry with Cu Kα radiation (λ = 1.54 Å). The range measured was 3–40° with steps of 0.04° (time per step 2 s) using a voltage of 40 kV and a current of 50 mA.

### Thermal analysis

Weight loss was determined by thermogravimetric analysis, using STA 6000 Simultaneous Thermal Analyzer (PerkinElmer Instruments, USA). Samples of 4–5 mg were analyzed at the heating rate of 20°C/min with the temperature range of 25–900°C. A N_2_ purge with a flow rate of 40 ml/min was used in the furnace.

Differential scanning calorimetry (DSC) was carried out with PYRIS Diamond DSC (PerkinElmer Instruments, USA). 30 µl aluminum pans with pierced lids were used to analyze samples of 1–3 mg. The heating rate of the samples was 10°C min^−1^, and the flow rate of N_2_ purge was 40 ml/min. Indium (156.60°C) was used to calibrate DSC system.

### High-performance liquid chromatography (HPLC)

Biochanin A and genistein were analyzed by HPLC (Hewlett Packard Series II 1090 LC instrument) from film formulations as samples of 4 cm^2^ in 25 ml of ethanol, using Inertsil ODS-3 5 µm, 150×4.0 mm + Security Guard Cartridge Kit (C18) 2.0×4.0 mm at 40°C. Biochanin A and genistein concentrations in buccal permeation experiments were analyzed by HPLC instrument Agilent 1100 Series (Agilent Technologies, Germany), using Discovery C18 5 µm, 150×4.6 mm column (Supelco Analytical, PA, USA) at 40°C. Biochanin A and genistein were determined by using slightly modified methods as presented by Han *et al.* [8] and Krenn *et al*. [29]. The eluent consisted of water adjusted with sulphuric acid to pH 2.7 and acetonitrile (58∶42 v/v%). The flow rate was 1.0 ml/min and the wavelength of detection was set at 254 nm. The injection volume of the samples was 10 µl. The retention times of biochanin A and genistein were 9.2 and 3.7 min, respectively. The linear concentration range of both compounds was established in the range of 0.1–100 µg/ml (R^2^ = 0.9999).

### Solubility analysis

To determine the solubility of biochanin A in ethanol, 20 mg of powder was added into 1 ml of ethanol and stirred at 700 rpm for 24 h at 37°C by using a Thermo shaker (BioSan, PST-100HL). The sample was centrifuged at 13000 rpm for 10 min and the supernatant was filtered (VWR, 0.2 µm pore-sized cellulose acetate sterile syringe filter), diluted and analysed for biochanin A content by using UV/Vis spectrophotometer (PerkinElmer, Lambda 25, USA).

### Permeability across buccal mucosa

Pig cheeks were obtained from a local abattoir within a few hours postmortem. Buccal mucosa was carefully separated from the muscle tissue with surgical scissors and stored at −20°C until further use. *In vitro* permeation studies of biochanin A from solution or film formulations were performed in Side-by-side-diffusion cells (Laborexin, Helsinki, Finland) or Franz diffusion cells (PermeGear Inc., PA, USA), respectively. Pieces of porcine full-thickness buccal mucosa were clamped between two halves of diffusion cells. The area of mucosa was 0.785 cm^2^ or 0.503 cm^2^ in studies with Side-by-side or Franz diffusion cells, respectively. To mimic physiological conditions, the cells were thermostated at 37°C by a surrounding socket or an incubator.

In studies with solutions, 3 ml of 1 mg/ml biochanin A solution in 0.1 M phosphate buffer (pH = 6.8) including 40% of DMSO was placed in the donor compartment of the diffusion cells. The same buffer was used in the receiver compartment, whose volume was 3 ml. 300 µl samples for HPLC analysis were collected from the receiver compartment and replaced by fresh buffer at 0.5, 1, 2, 3, 4, 5 and 6 h. In studies with films, 0.5 ml of 0.1 M phosphate buffer (pH = 6.8) was placed in the donor compartment of the diffusion cells to wet the buccal mucosa and the film (containing 2 mg of biochanin A). The same buffer was used in the receiver compartment, whose volume was 5 ml. After 4 h, both solutions were replaced with a phosphate buffer including 20% DMSO. 200 µl samples for HPLC analysis were collected from the receiver compartment and replaced by fresh medium at each time point.

### Data analysis

Unless otherwise stated in the text, all experiments were carried out as at least three replicates and results were expressed as mean and standard deviation. All further analyses, including linear regression analysis on the data from dose-response experiments and statistical testing was carried out using GraphPad Prism 4.0. software.

### Ethics

Pig cheeks used to supply the buccal tissue for permeability studies were obtained from a local abattoir from the material left unused within food production (HK Ruokatalo, Mellilä, Finland). No animals were specifically sacrificed for the needs of the study.

## Results

### Antichlamydial activity of isoflavones

Using a recently described high-content screening platform [30], biochanin A was identified as a hit from in-house library of 2000 compounds in our screen for antichlamydial compounds. In preliminary experiments with *C. pneumoniae* infections in human epithelial HL cells, no chlamydial inclusions were detectable in infected cultures treated with 50 µM biochanin A, indicating that biochanin A was able to completely block *C. pneumoniae* replication at this concentration. Experiments conducted using serial dilutions of biochanin A demonstrated a dose-dependent inhibition of *C. pneumoniae* inclusion counts, showing an IC_50_ value (the concentration yielding 50% of the inclusion counts compared to the untreated infection) of 12 µM (Table 2).

**Table 2:** IC_50_ values (µM) of isoflavones on *C. pneumoniae* and *C. trachomatis* inclusion counts.

Compound	*C. pneumoniae* IC_50_ (µM)*	*C. trachomatis* IC_50_ (µM)*
Biochanin A	12±5	62±9
Genistein	64±8	79±10
Genistin	>100	>100
Formononetin	53±10	-
Daidzein	87±12	-
Daidzin	>100	>100

*IC_50_ values were determined by treating the infected cell cultures with isoflavones at concentration 100, 75, 50, 25, 10 and 5 µM and determining the inclusion counts as described in Materials and methods. -  =  no inhibition. A statistically significant difference in *C*. *pneumoniae* IC_50_ values was observed between biochanin A and genistein (p<0.05, Student's t-test)

Our earlier studies have indicated that isoflavones genistein and daidzein are able to moderately suppress *C. pneumoniae* replication while the corresponding glycosides genistin and daidzin are not active at 50 µM [23]. Biochanin A differs chemically from genistein only by methylation of one hydroxyl group (Fig. 1) but this subtle change in the chemical structure resulted in a significant improvement in anti-*C*. *pneumoniae* activity. This feature was confirmed in re-testing of biochanin A and the four isoflavones reported in Alvesalo *et al*. [23] against *C. pneumoniae* (Table 2) and is also illustrated by the fact that none of the four previously studied isoflavones exceeded the hit limit even though they had been included in our screen together with biochanin A. As the current data suggested that methylation of the hydroxyl group improved the antichlamydial activity, we studied also formononetin, the corresponding methylated derivative of daidzein, which is also the other main isoflavone found in red clover [1]. The IC_50_ values presented in Table 2 demonstrate that biochanin A and formononetin are more potent in decreasing *C. pneumoniae* inclusion counts than their unmethylated analogues genistein and daidzein. However, also the hydroxyl group in position 7 (present in biochanin A and genistein but absent in formononetin and daidzein) contributes to the antichlamydial activity as can be observed from pairwise comparison of the IC_50_ values.

**Figure 2:**
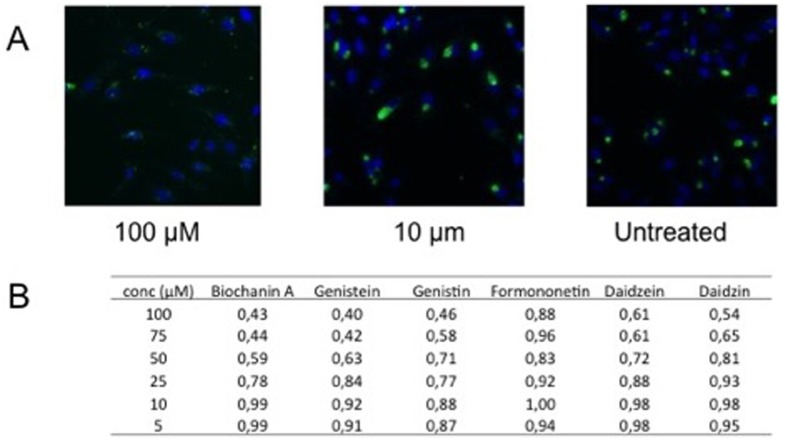
Effect of isoflavones on *C. trachomatis* inclusion size. A) Immunofluorescence images of *C. trachomatis* infected HeLa cells (untreated and treated with 100 or 10 µM biochanin A). *Chlamydia* inclusions are stained in green (polyclonal rabbit antibody raised against formalin fixed *C*. *trachomatis* elementary bodies [30]) and host cell nuclei are stained in blue with DAPI. B) Quantitation of average inclusion sizes in *C. trachomatis* infections treated with the isoflavones. Inclusion sizes are expressed as relative units proportional to the untreated controls. In a pairwise comparison of different concentrations, the mean inclusion size in biochanin A, genistein and genistin treated samples were statistically significantly smaller than in formononetin, daidzein and daidzin treated samples, respectively (p<0.05, Student's t-test).

Despite being members of the same taxonomical family, significant differences in susceptibility are known to exist between different *Chlamydia* spp. The effects of these six isoflavones were also investigated on *C. trachomatis*. As shown by the results presented in Table 2, biochanin A was the most potent of these compounds in decreasing *C. trachomatis serovar L2* inclusion counts, followed by genistein, while formononetin and daidzein were not able to decrease *C. trachomatis* inclusion counts with any of the concentrations tested (up to 100 µM). A similar activity pattern was observed also against C. *trachomatis* serovar K (data not shown). However, daidzein was able to moderately decrease the average *C. trachomatis* inclusion size at high concentrations while formononetin did not show antichlamydial effects even in this respect (Fig. 2). Biochanin A and genistein were significantly more active in decreasing inclusion sizes than daidzein, thus emphasizing the essential role of 7-hydroxyl substitution in the activity against *C. trachomatis*.

Taken together, biochanin A was the most efficient antichlamydial compound among the studied isoflavones. Cell viability assays conducted with resazurin reduction test confirmed that none of the six isoflavones assayed in this study decreased host cell (HL or HeLa) viability when applied in the same concentrations and exposure times as used for the antichlamydial assays (data not shown). Biochanin A was additionally assayed in different concentrations up to 500 µM on HL cells but no signs of decreased cell viability upon 72 h exposure were observed.

### Biochanin A as antichlamydial compound

**Figure 3:**
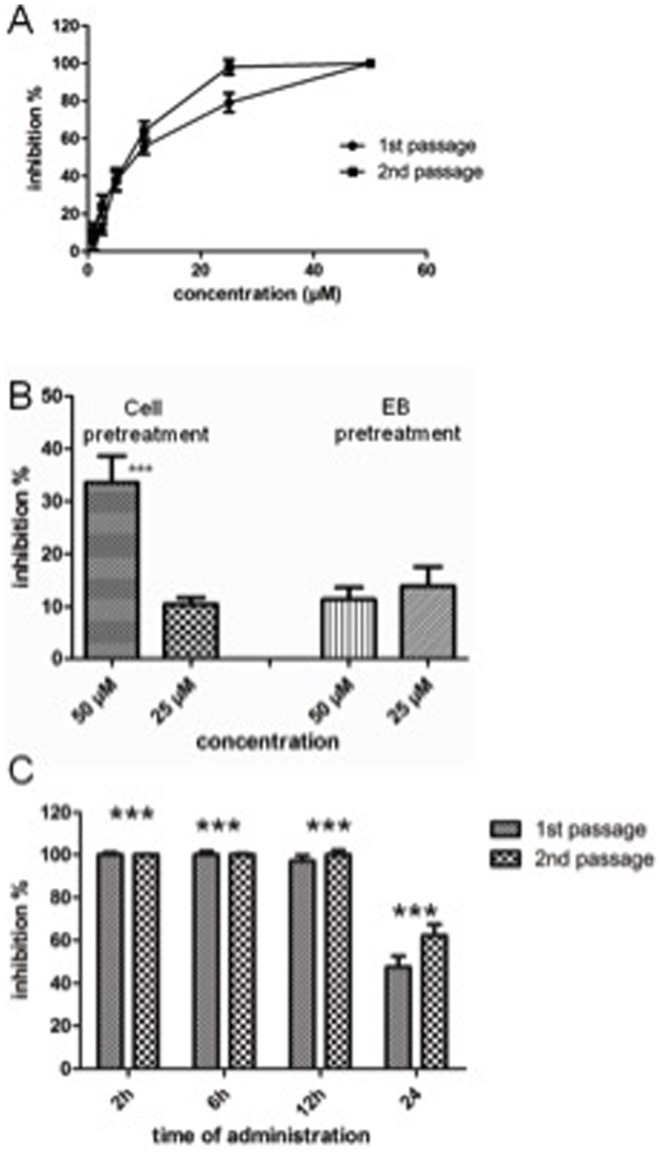
Impact of biochanin A on *C*. *pneumoniae* replication. A) Inhibition of *C. pneumoniae* inclusion counts and infectious progeny production by different concentrations of biochanin A. B) Effect of host cell or elementary body (EB) pretreatment with biochanin A on *C. pneumoniae* inclusion counts. C) Effect of delayed administration of biochanin A on the inhibitory capacity of biochanin A on *C. pneumoniae* inclusion counts (1^st^ passage) and infectious progeny production (2^nd^ passage). Biochanin A (50 µM) was added into infected cell cultures at 2, 6, 12 or 24 h post infection. In all experiments, the cultures were stained at 72 h as described in Materials and Methods. *** In B) and C) indicates statistically significant difference (*p*<0.05; unpaired t-test) compared to untreated control infection.

As the data presented above demonstrated that biochanin A is able to decrease *C. pneumoniae* and *C. trachomatis* infectivity measured as inclusion counts in infected cell cultures, we further characterized this isoflavone for its properties as *Chlamydia* growth inhibitor by an infectious progeny assay. Here, HL cells infected with *C. pneumoniae* were treated with biochanin A in concentrations ranging from 2.5 to 50 µM. At 72 h, lysates of the cultures were collected and used for infecting fresh HL cell monolayers to evaluate the number of infectious EBs produced in the biochanin A-treated cultures. As shown by the dose-response curves presented in Fig. 3A, production of infectious progeny EBs was efficiently inhibited by biochanin A and an IC_50_ value of 6.5 µM was achieved.

As obligate intracellular bacteria, *Chlamydia* have a unique biphasic replication cycle involving successive conversion between the elementary body (EB), the extracellular, infectious but nonreplicating form, and the reticulate body (RB), the intracellular replicating form [31]. Upon entry of an EB into its host cell, the complete life cycle resulting in the release of mature progeny EBs takes typically 72 h for *C. pneumoniae* in laboratory conditions, while most *C. trachomatis* strains can complete their life cycle in cell culture within 48 h. In standard antichlamydial assays with *C. pneumoniae*, the assayed compounds are added into infected cultures at 2 h post infection, i.e. when the bacterial inocula are removed from the wells. In order to further characterize effect of biochanin A on *C. pneumoniae*, a series of pretreatment and delayed administration time experiments were conducted.

Treatment of *C. pneumoniae* EBs with 50 or 25 µM biochanin A before using the EBs for inoculation did not have any significant effect on inclusion counts, indicating that biochanin A does not have a direct effect on this form of the bacterium (Fig. 3B, right). On the other hand, treating the HL cell monolayers with 50 µM biochanin A for 1 h prior to inoculation resulted in approximately 30% decrease in *C. pneumoniae* inclusion counts even though biochanin A was not added into the inoculation medium (Fig. 3B, left). This finding is in accordance with our earlier observation that pretreatment of HL cells with some flavonoids prior to *C. pneumoniae* infection decreases inclusion count even when the flavonoid is not added into the culture medium during the infection [23]. Flavonoids are known to accumulate to and interact with biological membranes [32], but the concentration of the remaining compound fraction after changing the medium is difficult to estimate.

In delayed administration time experiments, biochanin A (50 µM) was added into the culture medium at different time points post infection. Adding the compound to the infection medium according to the standard procedure immediately after the removal of inocula (2 h post infection) confirmed the 100% inhibition on inclusion counts and infectious progeny production by 50 µM biochanin A (Fig. 3C). Complete inhibition of inclusion formation and infectious progeny production was also observed when biochanin A was incorporated into the infected cultures at 6 or 12 h post infection, while adding the compound at 24 h reduced the number of visible inclusions by 47% and infectious progeny production by 62%. Furthermore, the inclusions seen in 24 h administration sample appeared significantly smaller than inclusions in the untreated control infections, indicating suppression of replicating bacteria in the inclusions. A similar activity pattern was observed also with *C*. *trachomatis* serovar K. Thus the antichlamydial activity of biochanin A is not limited to any specific step occurring early in the infectious cycle but rather the compound maintains its activity also in cases where the bacterium has already established its replicative machinery within the host cell.

### Permeability of biochanin A across buccal mucosa

**Figure 4:**
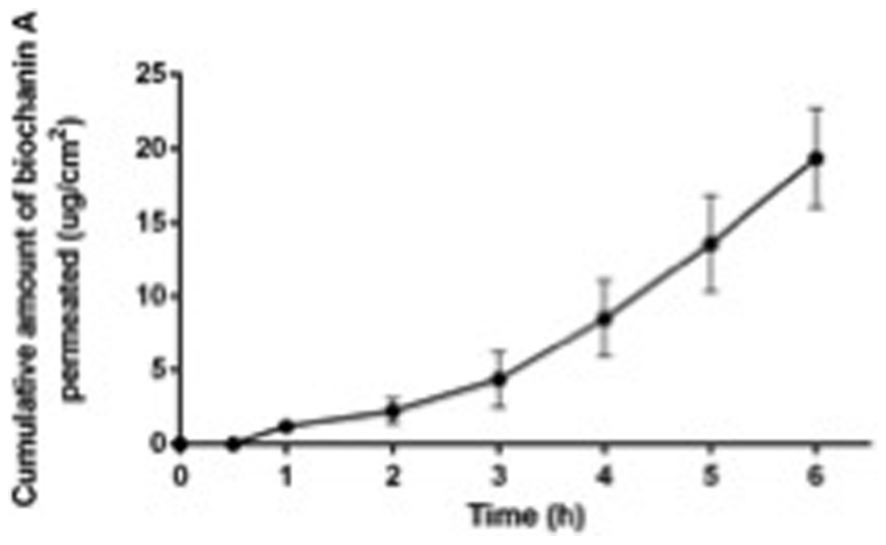
Permeability of biochanin A across porcine buccal mucosa. Cumulative amount of biochanin A was measured from the acceptor compartment of the diffusion cell.

Since our data indicated that already small chemical modifications seemed to have a remarkable impact on the antichlamydial activities of the related flavonoids, it was necessary to find an alternative route of administering biochanin A and avoid the demethylation reaction occurring during first pass metabolism. For this purpose, permeability of biochanin A across buccal mucosa was studied in a diffusion cell system using porcine buccal mucosa preparations. When biochanin A was added as 1 mg/ml in the donor compartment, 19.37±3.34 µg biochanin A was delivered across buccal mucosa per cm^2^ of tissue into receiver compartments in 6 h (Fig. 4). A steady-state flux of biochanin A was attained in three hours and the lag-time was 132 min. The steady-state flux was 5.00±0.83 µg/h·cm^2^. No detectable genistein peaks were observed in receiver compartment samples during 6 h of the permeation study. Furthermore, it has to be noted that in order to achieve HPLC-detectable amount of the permeated drug, the 1 mg/ml concentration of biochanin A in donor compartment was achieved by addition of DMSO. Even though the amount of biochanin A passed through in these conditions was rather small, the data showed a significant improvement compared to the intestinal bioavailability reports as demethylation did not occur.

### Preparation of buccal dosage forms

Encouraged by the lack of demethylation reactions during biochanin A permeation through buccal mucosa, it became feasible to look for suitable formulation for buccal administration. To this end, biochanin A containing buccal dosage forms were prepared by using two different film forming polymers: hydroxypropyl cellulose (HPC) and hydroxypropyl methylcellulose (HPMC) (Table 1). HPC is soluble both in water and ethanol, whereas HPMC is insoluble in pure ethanol, but soluble in ethanol-water mixtures. Biochanin A solubility in ethanol was determined to be 15 mg/ml which is more than 2000 times higher than the reported water solubility of this compound (7 µg/ml; [8]). Based on this, HPC was considered as the first-choice polymer to produce buccal films of different compositions from alcoholic polymer solutions or suspensions.

To improve film flexibility and reduce their brittleness, selection of a suitable plasticizer among the most commonly used ones (glycerol; propylene glycol, PG) and polyethylene glycol (PEG 400) was needed. Based on literature, HPC is soluble in PG and PEG 400, but insoluble in glycerol [33]. Based on the experiments conducted with PG and PEG, PG was chosen as plasticizer for the films, as films produced with PEG 400 were too soft. In addition, a disintegrant was selected with the aim to increase the release rate of biochanin A from the strip. Two superdisintegrants with different mechanisms of action, crospovidone and sodium starch glycolate, were studied for this purpose. In addition, maize starch was tested as disintegrant and hydrophilic bulking agent. Because these excipients are insoluble in cold water and ethanol; they were suspended in the film forming formulations. To further improve the bioavailability of biochanin A, different solubilizing agents such as nonionic PVP K30 and Tween 20, and anionic SDS were included in the film composition. Furthermore, *L*-menthol was used as a flavoring agent and odor enhancer due to its cooling sensation and peppermint taste.

**Figure 5:**
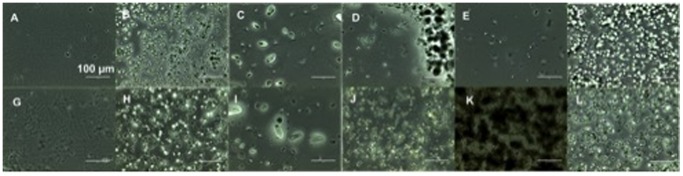
Optical microscope images of the film formulations. Top view: blank films (A–F), bottom view: Biochanin A containing films (G–L). Formulations: Faa (A,G), Fbb (B,H), Fdd (C, I), Fee (D, J), Fii (E,K) and Fjj (F,L) at magnification 40x.

The characteristics of the produced strips are presented in Table 3. The primary acceptance criteria for the prepared buccal films of different formulations were homogeneous appearance, ease of detachment from the intermediate liner, minimal stickiness and brittleness. Formulations containing SDS or PVP K30 as solubilizers (formulations Fkk and Fll) did not produce a detachable film. Different ratios of biochanin A to the solubilizers were studied, but none of these films prepared possessed proper characteristics. However, the other formulations gave films with acceptable properties (Fig. 5). The appearance of the produced strips containing biochanin A was similar to blank strips prepared without the API, except for formulation Fii (Fig. 5). In the cases when the casting formulations were of suspension type, the particles of the disintegrants could be observed with the naked eye (Fbb, Fdd, Fee, Fjj). Formulations Fbb, Fdd, Fee, and Fjj were stiffer than formulations Faa and Fii. The films produced from formulation Fii were totally matt if compared to the transparent blank film. Precipitation of biochanin A occurred, when the compound dissolved in ethanol was mixed with the aqueous HPMC solution as it was practically insoluble in water.

**Table 3:** Properties of sublingual films of biochanin A. Data are presented as mean ± standard deviation (^a^n = 12 and ^b^n = 3–4).

Form	Appearance	^a^Thickness (µm)	^b^Folding endurance	^b^Weight (mg/4 cm^2^)
Faa	Transparent, pale yellow, smooth surface, easy to detach	53±28	>500	16.5±1.3
Fbb	Slightly matt, pale yellow, easy to detach, rough surface, the lines of undissolved starch could be seen	66±32	>500	20.4±2.8
Fdd	Slightly matt, pale yellow, *not so* easy to detach, rough surface, the lines of undissolved disintergrant could be seen	57±27	>500	19.5±2.4
Fee	Slightly matt, pale yellow, easy to detach, rough surface, the lines of undissolved disintergrant could be seen	84±19	>500	21.4±2.7
Fii	Matt, pale yellow, smooth surface, easy to detach, produces cellophane noise during folding/bending	10±1	>500	5.9±0.5
Fjj	Slightly matt, pale yellow, easily detachable, the lines of undissolved starch could be seen	73±31	223±30	23.8±2.4
Fkk	Slightly matt, pale yellow, ductile, impossible to detach, the lines of undissolved starch could be seen	—	—	—
Fll*	Slightly matt, pale yellow, brittle, difficult-to-impossible to detach, the lines of undissolved starch could be seen	68±25	—	—

*analysis was done with small parts of the film (n = 4).

### Stability of biochanin A during formulation

The potential conversion of biochanin A to genistein in the prepared films was studied to ensure the release of the correct API from the formulations. An attempt to detect genistein in biochanin A powder and films prepared from biochanin A was made by using thermal analysis (S1 Figure). STA thermograms of biochanin A revealed the melting point at 212°C with subsequent degradation, whereas genistein showed the endothermic event at 303°C with further degradation. Thus, the presence of genistein in biochanin A containing formulation cannot be detected by STA, since biochanin A starts its degradation before the melting peak of genistein appears.

As an alternative approach, HPLC analysis was conducted to detect any traces of genistein in the biochanin A powder and the formulation Faa. Biochanin A powder contained less than 0.8% of genistein. The HPLC analysis of the film (formulation Faa) showed that the drug is present as biochanin A with the same percent of genistein as the pure powder (S1 Table).

### *In vitro* dissolution

**Figure 6:**
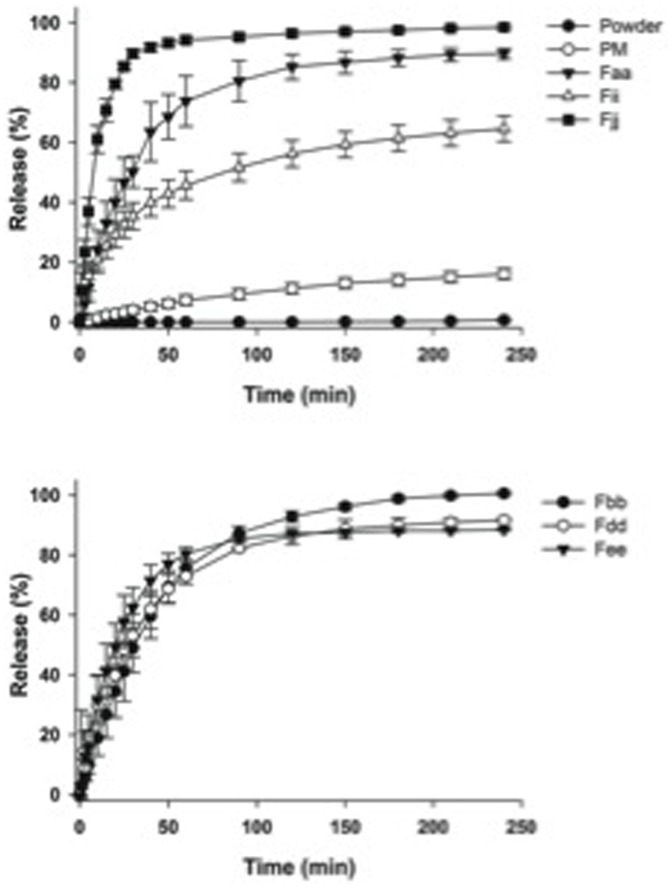
Dissolution profiles of biochanin A from buccal films of different composition, physical mixture (PM, corresponding to formulation Faa) and powder at pH 6.8. Data are presented as mean ± standard deviation (n = 3).

The release profiles of biochanin A from the prepared films, physical mixture and the pure powder are presented in Fig. 6. The amount of dissolved biochanin A powder was negligible, less than 2% in 4 h. The physical mixture of formulation Faa showed improved dissolution rate of biochanin A: 16% drug was released in 4 h. There was a drastic increase in the percent drug released by using a film of the same formulation (Faa): as more than 90% of the compound was released in 4 h. Dissolving biochanin A in the polymer-containing alcoholic solution before casting the strip gave rise to the production of the solid type films with improved dissolution characteristics. The improved bioavailability of some poorly soluble drugs from film preparations has been previously reported by other researches [34], [35]. However, the drug release from the film containing no solubilizers or disintegrants (formulation Faa) was relatively slow and incomplete. Considering the disintegrants crospovidone and sodium starch glycolate (formulations Fdd and Fee) did not affect the dissolution profile of biochanin A significantly. The addition of maize starch (formulation Fee) caused the complete release of biochanin A from the film in 4 h. Inclusion of Tween 20 as a solubilizer in the film formulation along with maize starch (formulation Fjj) fastened further the dissolution of biochanin A and caused complete release of the API. The release of biochanin A from the films made of HPMC instead of HPC (formulation Fii) was the slowest and incomplete due to partial recrystallization of biochanin A during film preparation as mentioned above. Additionally, the amount of HPMC present in the film was five times smaller than the HPC amount in the other films, due to the highest viscosity grade of the HPMC powder used. Solutions with higher HPMC concentrations were also tested but they were found to be difficult to prepare and almost impossible to cast uniformly. It is evident that adequate solubilisation of biochanin A is needed to guarantee fast release of it from the solid formulation.

To classify the films between immediate- or controlled-release formulations, the film of formulation Fjj (HPC containing maize starch and Tween 20) is of immediate-release type solid dosage form as it released 85% of biochanin A in less than 45 min. The films with other formulations can be regarded as modified-release solid dosage forms.

### Permeation of biochanin A released from the films across buccal mucosa

Buccal mucosa permeability studies were also extended on biochanin A released from Fjj films, indicating a behavior consistent with the earlier permeability data. When dissolved in phosphate buffer, no biochanin A had permeated across the buccal mucosa after 4 h. The medium was then changed to a buffer with 20% DMSO in order to increase the solubility of biochanin A in the medium and therefore to increase its flux across the mucosa. In 20 h in the new buffer 21.19±4.74 µg biochanin A was delivered across buccal mucosa per cm^2^ of tissue into receiver compartments of the Franz cells. A steady-state flux of biochanin A was attained in one hour after introducing DMSO into the system. The steady-state flux was 1.10±0.26 µg/h·cm^2^. No detectable degradation peaks (genistein or other) were observed in receiver compartment samples during the permeation study.

### Solid state characterization

**Figure 7:**
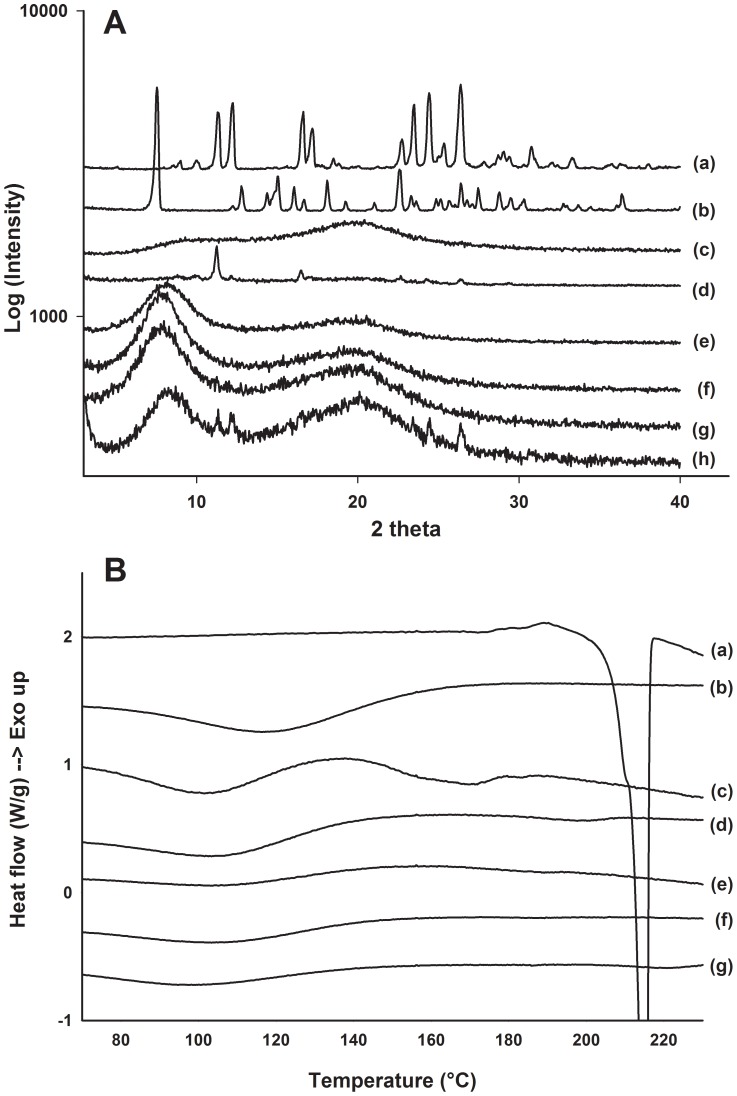
X-ray diffraction and DSC measurements of film formulations. A) X-ray diffraction patterns of (a) biochanin A powder, (b) genistein powder, (c) HPMC powder, (d) film Fii, (e) blank film Faa without biochanin A, (f) film Faa, (g) film Fjj and (h) physical mixture of formulation Faa. B) DSC thermograms of (a) biochanin A powder, (b) HPMC powder, (c) film Fii, (d) blank film Faa without biochanin A, (e) film Faa, (f) film Fjj and (g) physical mixture of formulation Faa.

As indicated by the optical microscope images in Fig. 5, biochanin A powder is a crystalline material. To study the physical state of biochanin A in the films, X-ray diffractograms were collected from samples with different formulation. The X-ray diffractograms of the film formulations Faa and Fjj showed a halo pattern, while biochanin A-related peaks were observed from the samples of physical mixture and HPMC-based formulation Fii (Fig. 7A). These data indicated that the improved release rate of biochanin A from films Faa and Fjj was due to the presence of biochanin A in noncrystalline, amorphous state in these films. This was also in agreement with the visualization and dissolution data (Fig. 5 and Fig. 6).

To further address this question, differential scanning calorimetry (DSC) experiments were carried out. Thermograms of the pure biochanin A powder showed a melting endothermic event at 212°C, while none of the biochanin A containing films showed any melting events for biochanin A (Fig. 7B). Also in the case of formulation Fii and physical mixture of formulation Faa components, no indication of crystalline form of biochanin A was seen in the thermograms even though X-ray diffractograms indicated its crystallinity. Probably, softening of the polymer at elevated temperature and presence of propylenglycol were responsible for dissolution of biochanin A in the formulations before its melting point.

## Discussion

Rather than as a bacteriocidal or bacteriostatic agent, biochanin A has been discussed as a potentiator of other antimicrobial agents. Biochanin A, as well as two other isoflavones genistein and orobol, have been reported to potentiate the antibacterial activities of norfloxacin and a natural plant antibiotic berberine against *Staphylococcus aureus* at concentration of 6.25 mg/l (approximately 22 µM), but none of these three isoflavones as such suppressed *S. aureus* growth even at the highest concentration tested (100 mg/l; approximately 350 µM) [36]. Similar behavior has also been reported against *Mycobacterium smegmatis*: biochanin A had a MIC value of 256 mg/l (approximately 900 µM) for growth inhibition, but was able to potentiate the antimycobacterial effects of other chemical agents at significantly lower concentrations (10–32 mg/l, i.e. 35–110 µM, respectively) [37]. Based on mechanistical studies, such potentiating effects have been linked to inhibition of bacterial multidrug resistance (MDR) efflux pump functions by biochanin A.

The results presented in the current work demonstrate that besides its potentiating effects on other antibacterial compounds, biochanin A directly inhibits the growth of Gram-negative intracellular bacteria in genus *Chlamydia*. According to our data, it completely prevents the formation of *C. pneumoniae* inclusions at concentrations of 25 µM or higher and significantly decreases the inclusion counts at low micromolar concentrations. It also prevents the formation of new infectious progeny, which is seen as the lack of infectivity of the collected progeny upon new infection cycle. Furthermore, treatment of the *C. trachomatis* infected cell cultures with biochanin A significantly suppresses inclusion counts and decreases the mean bacterial inclusion size.

The current work represents to our knowledge the first report of biochanin A or other isoflavones as direct growth inhibitors of Gram-negative bacteria. The success rates of discovering antibacterial compounds from plants are typically significantly lower than from microbial sources, and identification of plant-derived compounds active against Gram-negative bacteria has been particularly challenging [38]. Given the atypical nature of plant derived compounds as antibacterial agents and the essential contribution of host cell metabolism to the intracellular replication process of *Chlamydia*, it is not clear whether the antichlamydial activities of isoflavones are mediated through a bacterial target or via modulation of the host cell in a manner unfavorable to the bacterium. Pretreatment experiments demonstrated that biochanin A does not affect *C. pneumoniae* in its extracellular (EB) form but the fundamental changes in chlamydial morphology and metabolism upon its entry to a host cell offer a spectrum of possible targets for growth inhibition. Considering host cell targets, any effects on host cell protein expression can be ruled out due to the presence of cycloheximide in the infection medium. Potential targets are thereby limited to the bacterium itself or a host cells component present at the time of inoculation.

Besides its affinity to bacterial efflux pumps, biochanin A has been shown to block also p-glycoprotein, the major MDR efflux pump in mammalian cells which is structurally unrelated to the bacterial efflux pumps. Altered p-glycoprotein substrate drug transport and bioavailability upon coadministration with biochanin A has been reported both in vitro and in vivo [39], [40]. Chemical inhibition of MDR1 was recently shown to restore the antibiotic susceptibility of *C. trachomatis* in hypoxic conditions, but the MDR1 inhibitor cyclosporine A used in the study did not have any significant effect on *C. trachomatis* growth when used alone [41]. These findings further support the view that the affinity of biochanin A to bacterial and mammalian efflux pumps may be an additional beneficial feature for the antibacterial applications with this compound although in the light of the current evidence it is not the main mechanism of action for the direct antichlamydial effects.

In our earlier studies, we have identified several other flavonoids as *C. pneumoniae* growth inhibitors [23], [25]. The small structure-activity relationship analysis conducted in this work confirms our earlier observations on the importance of 5,7-dihydroxyl substitutions for efficient anti-*C*. *pneumoniae* activity. However, biochanin A differs from the earlier identified highly active antichlamydial flavonoids by its isoflavone structure. Furthermore, the current work expands the antichlamydial activities of isoflavones to cover also *C. trachomatis*. *C. pneumoniae* and *C. trachomatis* are both human pathogens but they differ significantly in their tissue tropism and diseases they are able to cause. The degree of functional conservation between *C. pneumoniae* and *C. trachomatis* is relatively high and especially the functions related to transcription and metabolism are well conserved between the two species. However, comparative analysis of the genetic sequences of these two bacterial species has revealed also essential differences between the two species [42], which is also evident within the differences in antibiotic susceptibility between *C. trachomatis* and *C. pneumoniae* [43]

Due to the interest in isoflavones as phytoestrogens, a significant body of information is available on their pharmacokinetic properties. In healthy adults, peak plasma concentrations are typically reached within 5–9 h after ingestion [6]. The efficiency of intestinal absorption is, however, limited by the extensive first pass metabolism and recycling. As discussed earlier, majority of biochanin A is converted to genistein prior to its absorption via demethylation reactions and less than 5% of biochanin A in bloodstream is found in its parent methylated form after ingestion of the pure compound.

Given these challenges in oral bioavailability and the weaker antichlamydial activity of genistein compared to biochanin A, buccal route of administration was studied as an alternative means for delivering biochanin A into the body. Although data with Caco-2 cells and in perfused intestine models show that biochanin A is permeable through intestinal membranes [9], buccal mucosa is known to possess properties different from intestinal wall in regards of epithelial cell organization, lipid composition and biotransformation [44]. The permeability studies carried out with porcine buccal mucosa demonstrated that biochanin A is able to penetrate buccal mucosa and conversion of biochanin A to genistein was not observed during the process. Similar results were obtained from the preliminary permeation studies of biochanin A released from Fjj films in Franz diffusion cell setup.

Permeation of biochanin A across the buccal mucosa was dependent on the concentration of the dissolved drug since the concentration difference between two sides of the mucosa serves as driving force for drug diffusion. Thus an increase in the concentration of the dissolved biochanin A would provide higher flux leading to faster permeation. It is obvious that the formulated films provided faster dissolution rates, while the solubility of the biochanin A in water however remains the same, leading to permeation rate to remain constant. Based on these observations, it was concluded that the rate-limiting step on biochanin A buccal bioavailability was permeation rather than dissolution. While addition of a permeation enhancer into the film formulation might thus allow full exploitation of the increased dissolution rate of biochanin A, these data demonstrate that biochanin A can be absorbed via buccal route without conversion to genistein or other metabolites.

Drug delivery by means of oral films is a rapidly growing research and marketing area [34], [35], [45]–[49]. The advantages of the thin films made of water soluble polymers can include, among other things, improved bioavailability of poorly soluble drugs, reduction in the dose and drug entrance of the systemic circulation without undergoing first-pass metabolism. These features make this type of the delivery system potentially feasible for developing the biochanin A containing formulation to maximize compound's effect on the bacteria and minimize its dose.

Oromucosal film preparations provide a feasible route of administration for biochanin A. During our dissolution studies, we observed significant differences in dissolution rates between the formulations. The film containing hydroxypropyl cellulose as a film-forming polymer, Tween 20 as a solubilizer, and maize starch as a disintegrant was found to show the fastest release profile. Thus, maize starch was found to work better than the two tested superdisintegrants. In the case of crospovidone, disintegration of the formulation occurs by capillary action and hydration [50], whereas pronounced swelling occurs when sodium starch glycolate is used [51].

The in vitro dissolution studies showed that by film formulations, a significant improvement of the dissolution rate of biochanin A was achieved without the need for chemical modifications of the active ingredient. Characterization of the formulations indicated that increased dissolution rate was achieved due to the solubilizing effect of the hydrophilic additives and by the presence of biochanin A in amorphous state in the formulation. This finding is also supported by another recent report on the preparation of solid dispersions with amorphous biochanin A, using hydrophilic polymers Solutol HS 15 and HPMC 2915 [8]. With these formulations intended for conventional oral administration, the bioavailability was improved by 8- to 60-folds in comparison to unmodified biochanin A powder. However, ethanol - dichloromethane mixture of 1∶1 was used as a solvent to dissolve biochanin A and the ethanol-insoluble HPMC. Due to toxicity of dichloromethane, its use during preparation of solid dosage forms is undesirable and thus the HPC-based formulation used in this study, requiring only ethanol, is a more attractive alternative, and the buccal administration route optimally provides added value to administration of the antibacterial compound through the avoidance of first-pass metabolism.

In summary, the current work demonstrates that biochanin A is a potent inhibitor of the intracellular gram-negative bacteria *Chlamydia pneumoniae* and *C. trachomatis*. According to our cell viability assay results, it shows no harmful effects on the host cell viability and the safety of biochanin A for human use is supported also by the routine intake of biochanin A and related isoflavones in foods and dietary supplements. The formulated films showed significantly improved dissolution rate of biochanin A compared to the powder or a physical mixture, presumably due to the solubilizing effect of the hydrophilic additives and presence of biochanin A in the amorphous state. Biochanin A was released from film formulation and could permeate porcine buccal tissue without changing to genistein. From a natural product drug discovery perspective, these data open new alternatives for improving the bioavailability of similar compounds without the need for chemical modifications, which often affect the desired biological activity.

## Supporting Information

S1 Figure **Simultaneous thermal analysis (STA) of biochanin A and genistein powder.**
(TIFF)

S1 Table **HPLC analysis of biochanin A and genistein powder and formulation Faa in ethanol.**
(DOCX)
