# Supplementary material for: Inhibitory Activity of the Isoflavone Biochanin A on Intracellular Bacteria of Genus Chlamydia and Initial Development of a Buccal Formulation
Source: PLoS One. 2014 Dec 16;9(12):e115115. doi: 10.1371/journal.pone.0115115 (PMC4267780; doi:10.1371/journal.pone.0115115)
Supplement: S1 Table — HPLC analysis of biochanin A and genistein powder and formulation Faa in ethanol. (DOCX) [file pone.0115115.s002.docx]

|  |  | **Biochanin A** | | | **Genistein** | | |
| --- | --- | --- | --- | --- | --- | --- | --- |
|  | **mg/ml** | **Retention (min)** | **Area** | **Height** | **Retention (min)** | **Area** | **Height** |
| **Biochanin A** | 0.1 | 11.6 | 5704.2 | 300.7 | 3.8 | 43.3 | 5.3 |
| **Genistein** | 0.1 | -- | -- | -- | 3.8 | 6467.2 | 792.9 |
| **Biochanin A Genistein** | 0.05  0.05 | 11.6 | 2838.9 | 148.4 | 3.8 | 3250.0 | 396.6 |
| **Faa** | 0.2 | 11.8 | 11193.9 | 591.4 | 3.9 | 56.9 | 7.1 |

**Table S1.** HPLC analysis of biochanin A and genistein powders and formulation Faa in ethanol.
